# Decision of the Supreme Court of the United States in the Rubber Case

**Published:** 1873-05

**Authors:** 


### EDITORIAL, ETC.

---

*Decision of the Supreme Court of the United States in the Rubber Case.*—In the April No. of *Journal* we informed our readers that Dr. S. S. White, through his counsel, Messrs. Jeremiah S. Black and Henry Baldwin, Jr., Esq's, had succeeded in having set aside the decree of the Supreme Court on the ground of collusion, &c., and thus saving the profession from what they consider an unjust burden. The case is therefore again open for the profession to establish by proof the invalidity of the Cumming's patent. The following from the April No. of the *Dental Cosmos* is the decision of the Supreme Court rendered March 3rd, 1873, and for which the profession owe a debt of gratitude to Dr. S. S. White, as it was in his behalf and through his efforts that the appeal or decision was set aside and the case again opened :

SUPREME COURT OF THE UNITED STATES.

No. 133. December Term, 1871.

Benoni E. Gardner, Appellant, *versus* The Goodyear Dental Vulcanite Company, *et al.*

*Appeal from the Circuit Court of the United States for the District of Rhode Island.*

*On Motion.*

Mr. Chief Justice Chase delivered the opinion of the Court.

The original suit in equity was brought by the Goodyear Dental Vulcanite Company against Gardner to enjoin him from the use of certain patented subjects belonging, as alleged, to the Company, and for an account. The case was heard upon a bill, answer, and testimony, and there was a decree in favor of the Company in the Circuit Court for the District of Rhode Island, in September, 1870. Upon appeal to this court the decree below was affirmed on the 6th of May, 1872, but the opinion has not been read. The defense was conducted by counsel originally employed and paid by Newbrough, under whom Gardner was licensee. On the 1st of July, 1869, before the decree in the Circuit Court, Newbrough and the Company compromised all matters of difference between them, with the understanding that this suit should go on to the final hearing and determination both in the Circuit Court and in this court on appeal, as if the compromise had not been made.

The Company, however, paid the counsel employed for the defense as well as for themselves in Circuit Court, and subsequently in this court. These facts appear from the record and from the admissions of the Company in the ninth article of their answer to the motion to dismiss the appeal. They are the only facts which we think it necessary to notice.

It may be that the Company has not become the legal or equitable owners of the opposing interests involved in the suit. There may be, and doubtless are, large opposing interests of which they are neither the legal or equitable owners. But it cannot be admitted that one party to a suit can pay the fees of counsel on both sides, both in the court below and on appeal, without being held to have such control over both the preparation and argument of the cause as to make the suit merely collusive in both courts. It can make no difference that the counsel fees were charged to the party apparently though not really liable to pay them, and payment from the other party procured through him. This, indeed, is a circumstance against the party who pays the fees rather than in his favor.

The motion to vacate the decree of affirmance heretofore made, to dismiss the appeal, must, therefore, be granted, and an order made to recall the mandate which has been issued to the Circuit Court. We take occasion, however, to say that we see nothing in the conduct of the counsel who actually represented the Company which merits blame, or which ought to affect in any degree the high esteem in which they have been held. Neither of them appears to have had any knowledge of any arrangements made by their client with the opposing party.

True copy.

[SEAL.] Test: D. W. MIDDLETON,

*Clerk Superior Court, U. S.*

[That there may be no question as to the scope and effect of the foregoing decision, we have procured and append a copy of the mandate which had been issued to the Circuit Court which is recalled and annulled by the decree on the motion to dismiss the appeal.]

UNITED STATES OF AMERICA, ss.

THE PRESIDENT OF THE UNITED STATES OF AMERICA.

*To the Honorable the Judges of the Circuit Court of the United States for the District of Rhode Island, greeting:*

*Whereas*, lately, in the Circuit Court of the United States for the District of Rhode Island, before you, or some of you, in a cause between The Goodyear Dental Vulcanite Company and Josiah Bacon, complainants, and Benoni E. Gardner, respondent, wherein the decree of the said Circuit Court entered in said cause on the 8th day of September, A. D. 1870, is in the following words, viz.:

"Upon the coming in of the master's report, and on motion of the complainants' counsel, it is ordered and decreed by the Court that the said report be confirmed and established, and that the said defendant, Benoni E. Gardner, and his agents and servants, be perpetually enjoined from making, using, or vending to others to be used, any one or more dentures or plates for artificial teeth manufactured according to the process described in the reissue patent, in said bill of complaint mentioned.

"And the Court doth further order and decree that the said defendant do forthwith pay unto the complainants the sum of seventy-five dollars, being the amount of profit found by the said master's report to have been received by the said defendant, Gardner, from the manufacture and sale of plates for artificial teeth manufactured according to the process described in the said reissue patent, in violation of the exclusive right of the complainants.

"And the Court doth further order and decree that the said defendant, Gardner, do pay to the said complainants the costs of this suit, taxed at two hundred and forty-eight dollars and eighty-four cents ($248.84), entered as the decree of this Court, per order thereof, this 8th day of September, A. D. 1870."

As by the inspection of the transcript of the record of the said Circuit Court, which was brought into the Supreme Court of the United States by virtue of an appeal, agreeably to the act of

Congress, in such case made and provided, fully and at large appears.

*And Whereas*, in the present term of December, in the year of our Lord one thousand eight hundred and seventy-one, the said cause came on to be heard before the said Supreme Court, on the said transcript of the record, and was argued by counsel: On consideration whereof, it is now here ordered, adjudged, and decreed by this Court that the decree of the said Circuit Court in this cause be and the same is hereby affirmed, with costs and interest until paid, at the same rate per annum that similar decrees bear in the courts of the State of Rhode Island, and that the said complainants recover against the said respondent, Benoni E. Gardner, three hundred and ninety-nine dollars and nineteen cents for their costs herein expended, and have execution therefor. 6*th May*, 1872.

You, therefore, are hereby commanded that such execution and proceedings be had in said cause, as according to right and justice, and the laws of the United States, ought to be had, the said appeal notwithstanding.

Witness the Honorable Salmon P. Chase, Chief Justice of said Supreme Court, the first Monday of December, in the year of our Lord one thousand eight hundred and seventy-one.

Costs of complainants: Clerk, $379.19, Attorney, $20.00. Total, $379.19.

Taxed by D. W. MIDDLETON,
*C. S. C. U. S.*

SUPREME COURT U. S. DECEMBER TERM, 1871.

Costs of the Goodyear Dental Vulcanite Company *et al.*, in No. 133, 1870.

$379.19; Fee book L; pa. 378; Test.: D. W. MIDDLETON,
*C. S. C. U. S.*

1872, May 14. Received payment, per A. POLLOK, Esq.
D. W. MIDDLETON,
*C. S. C. U. S.*

### THE BENONI E. GARDNER APPEAL CASE
### IN THE
### SUPREME COURT OF THE UNITED STATES.

"The evidence in a suit, when only one party is heard, is straighter than a line." *Hindu Proverb.*

In the June number of the *Dental Cosmos* we endeavored correctly to exhibit the procession of events and the persons concerned in the decision obtained in the above case in the Supreme Court. We quote the conclusion of that article:

"What all this brings us to in the present relation of dentists to vulcanite work and the patent which controls it. The Goodyear patent expired May 6th, 1872. On that day this decision was announced, which gives to the Cummings patent a standing in court which deprives individual dentists of any hopeful chance of resisting them.

"There is naturally due to a decision of the Supreme Court of the United States a certain weight and effect which this one will fail to secure permanently. We charge that the parties by whom this case has been made up have committed a deliberate contempt upon this highest tribunal of our country; that they purchased this case, so as to control it; that since its purchase, they have directed both sides of it, for the express purpose of gaining a decision rapidly and safely. Reckless of the dignity of the courts through which they have worked; reckless of the interests of thousands of dentists whom they plotted to entrap in meshes from which they now represent that there can be no escape; reckless of the professional honor of their own ablest advocate; reckless of everything but the success of their plans, and their selfish greed for a harvest of plunder.

"We charge that in this case, since the settlement of the Goodyear Dental Vulcanite Company with John B. Newbrough, July, 1869, there has been no veritable plaintiff, because they were bound by that settlement to give a full license to the man whom they are, in this suit, pretending to sue for infringement. There has been no veritable defendant, because B. E. Gardner was licensed, and protection for him secured in Newbrough's contract.

"We charge that this case has, ever since July, 1879, been collusive, and that it is a fraud upon the courts, and upon those against whom this decision is now sought to be enforced, and has never had any color of verity, except that which it obtained through the misguided efforts of those dentists who aided in giving it that semblance by their action just previous to and at the hearing.

"Such a decision—a decision so obtained—is not entitled to, and will never receive, the deference and respect of intelligent, law-abiding, and equity-loving citizens.

" The proofs of collusion and fraud, which have gathered and hold, are safe beyond the possibility of concealment or purchase. They are ready to aid whatever resistant action the dentists of the United States may decide upon or be advised to take; whether that shall be to seek a rehearing of this badly prepared case, or to claim a fair hearing on a new one.

" Be sure that when presented, the Court will listen to them, and that this decree of May 6th, 1872, will NOT BE FINAL. We advise our friends not to believe it, not to submit to it, nor to think of it as FINAL. Dentists should cease the use of rubber, and refuse to take or pay for licenses. Half-way measures will not do now. The refusal must be absolute, and it must be general. Let us test the question whether there is in this great body of educated men enough self denial to make this course a success.

" If this feeling and determination is established in the minds of individuals, it will be communicated from one to the other, extend to local meetings and conventions, and become accepted and recorded as the decision of these primaries.

" Out of these a league may be formed, and a national organization set up, which will exert an influence strong enough to draw in the wavering, and those who have calculated upon making profit out of the abnegation of their brethren.

"Ultimately, with a careful selection of leaders, wise forethought, the best and ablest counsel in all the land, and the abundant means which a very small contribution of a host of members will aggregate, this Goodyear Dental Vulcanite Company shall suffer first the extinction of their revenue, and then a trial, which will extinguish the title of their Cummings patent."

Convinced of the truth of the foregoing charges, and seeing how much oppression by fraud might be perpetrated under this decree of the Supreme Court, we thought it our duty to inform the Court of the facts, and procure a rescission of its decree, and thus save the profession from the necessity of passing under a yoke prepared for them.

Accordingly, we sought the advice and aid of counsel, who, upon examination of the case, assured us that the law could not thus be evaded and prostituted by corrupt men. In the interest of the dental profession not parties to the suit, but whose rights might be seriously affected, we caused the facts to be laid before the Supreme Court of the United States, and asked that the decree should be stricken out and the appeal dismissed, on the ground of the collusion and fraud before mentioned. The Court saw how it had been imposed upon, and granted our prayer.

The fact that the Company in question resorted to foul play shows that they themselves were conscious of having a bad hand; otherwise they would not have relied upon "ways that were dark" and tricks that turned out to be vain.

We have given a full copy of the record of the proceedings on our motion, to verify our statement, and to show that we charged *before* this motion was made has been proven. To the mind of a candid reader, we think the record exhibits in a most unenviable position the parties concerned; but if it furnishes any ground for charity, we give them the benefit of it.

This decree, in which Mr. Justice Clifford himself concurs, negatives, neutralizes, and nullifies the effect of the decree of the Circuit Court of Rhode Island, supersedes the impending affirmance of that decree by the Supreme Court, and thus obviates the difficulties which would otherwise have been in the way of any subsequent or further endeavors by the profession to obtain a hearing upon the real merits of the case.

It is sufficient to say that all questions have been, or are, or may be, material or relevant in connection with the Cummings Patent, and the rights of the profession in view of that patent, are now and thus open and free for discnssion by and in behalf of the profession, for establishment by proof, for argument by counsel, and for adjudication by the courts.

Of our own course in connection with this appeal, the action of the Supreme Court of the United States is sufficient explanation and vindication, correcting as it must all misapprehensions, neutralizing all misrepresentation, and showing in the light of a complete triumph to what interest our efforts were devoted, and with what effect. Working alone, and assuming the sole and entire responsibility, we resorted to the only means left available to foil the well-planned scheme of that appeal, by laying before the Court, immediately upon the opening of the adjourned term, the facts which have led them to the decision that now takes the place of the affirmance that would otherwise have been promulgated. This affirmance, once promulgated, would have rendered it virtually impracticable to reinstate the profession in the advantageous position which it now occupies. The proceeding itself, and its result, are without precedent in the history of the Supreme Court. Never before has there been a case in which an appeal was fully argued, a decision made, a decree affirmed, and the affirmatory mandate issued, and then the decree of affirmance vacated, the opinion withheld, the appeal dismissed, and the mandate recalled, upon the ground that the suit, both below and above, was "MERELY COLLUSIVE."

SAMUEL S. WHITE.

